# Precancerous Lesions of the Head and Neck Region and Their Stromal Aberrations: Piecemeal Data

**DOI:** 10.3390/cancers15082192

**Published:** 2023-04-07

**Authors:** Ashlee Harris, Thomas Andl

**Affiliations:** Burnett School of Biomedical Sciences, University of Central Florida, 12722 Research Pkwy, Orlando, FL 32826, USA; ashlee.harris@knights.ucf.edu

**Keywords:** tumor stroma, head and neck cancer, precancerous lesions, oral potentially malignant disorders (OPMD), squamous cell carcinoma, inflammation, cancer-associated fibroblasts, chemoprevention

## Abstract

**Simple Summary:**

Advanced stage at presentation, limited treatment options, and high recurrence rates are the main factors contributing to poor outcomes for patients with head and neck squamous cell carcinoma (HNSCC). However, these tumors develop from precancerous lesions that may be more susceptible to treatment. All previous efforts to intervene at the precancerous stage have failed. Recently, researchers have turned their attention to the role of the stroma, the tissue surrounding the cancer cells, in the transition from precancer to cancer. This review aims to summarize our current understanding of stromal changes from precancer to cancer, with the goal of identifying new opportunities for chemoprevention to improve patient outcomes.

**Abstract:**

Head and neck squamous cell carcinomas (HNSCCs) develop through a series of precancerous stages from a pool of potentially malignant disorders (PMDs). Although we understand the genetic changes that lead to HNSCC, our understanding of the role of the stroma in the progression from precancer to cancer is limited. The stroma is the primary battleground between the forces that prevent and promote cancer growth. Targeting the stroma has yielded promising cancer therapies. However, the stroma at the precancerous stage of HNSCCs is poorly defined, and we may miss opportunities for chemopreventive interventions. PMDs already exhibit many features of the HNSCC stroma, such as inflammation, neovascularization, and immune suppression. Still, they do not induce cancer-associated fibroblasts or destroy the basal lamina, the stroma’s initial structure. Our review aims to summarize the current understanding of the transition from precancer to cancer stroma and how this knowledge can reveal opportunities and limitations for diagnostic, prognostic, and therapeutic decisions to benefit patients. We will discuss what may be needed to fulfill the promise of the precancerous stroma as a target to prevent progression to cancer.

## 1. Introduction

The observation that cancer cells are surrounded by a specialized stroma is as old as tumor biology itself (excellently reviewed in [1]). Virchow is often credited with being the first to describe the peculiar stroma of tumors, especially its unusual inflammatory component. Virchow connected this cancer-associated inflammation in his “irritation theory” (“Reiztheorie”) and proposed that chronic inflammation may cause cancer [2,3]. The idea of a key role of inflammatory cells in cancer received renewed interest in recent years, but its focus has switched to the “reactive stoma” as a part of the tumorigenic process and a target for immunotherapies [4].

But this renewed interest in the tumor stroma also highlights the fact that the relevance of the tumor stroma had been basically ignored until the 1980s. Perhaps one of the earliest and most profound additions to our understanding came in the early 1970s when Folkman explored a fundamentally stromal event, i.e., angiogenesis in the context of cancer [5,6,7]. This eventually resulted in the concept that tumors resemble never healing wounds and that the stroma plays an integral part in tumorigenesis [8]. The realization that the stroma, including the non-cellular components of the extracellular matrix, regulate tumor cell behavior has revolutionized tumor biology [9]. In time, the cells that synthesize the majority of the tumor extracellular matrix were identified as myofibroblasts [10,11,12].

The emergence of the tumor stroma as a complex and integral part of the tumor mass resulted in the mid-1980s in the approval of treatments based on targeting the tumor stroma (e.g., 1986 IFNA2 treatment), including today’s immunotherapies.

Nevertheless, all this progress in understanding tumor biology and the role of the stroma in tumor progression and treatment resistance has only mildly improved patient survival. This may be due in part to the underestimated complexities and flexibility of the cancer cell–stroma interactions.

One particularly under-investigated area is precancer, the state before the epithelial cells take full control of the stroma and mold it to their own advantage. In the last two decades, the usefulness of the terms “precancerous” and “premalignant” have been intensively discussed in the field of oral cancer. Ultimately, these terms have been abandoned in favor of the concept of “oral potentially malignant disorders” (OPMDs) [13] to describe our inability to predict which lesions can progress to cancer and which ones will fail. Therefore, OPMDs represent the collective of lesions that are macroscopically (e.g., leukoplakias, erythroplakias) and microscopically (e.g., hyperplasia, dysplasia, carcinoma in situ) present in patients and may have the potential to advance to squamous cell carcinoma (SCC). The confusion in terminology and lack of clear definitions highlights that these lesions are extremely difficult to categorize, to use for prognosis, and to prevent from progressing to OSCC. For this review, we refer to this group of lesions in the head and neck region as potentially malignant disorders (PMDs) because we discuss lesions beyond the oral cavity.

PMDs have been the target of several unsuccessful chemopreventive efforts to tackle head and neck squamous cell carcinoma (HNSCC) formation and the prevention of secondary primary tumors. The longstanding efforts to intervene in the transition to cancer highlights the clinical problem of “precancer” in HNSCC biology. Due to the prolonged and spatially broad exposure to carcinogens in the head and neck area, large areas of the epithelium are genetically altered and represent various states along the normal to cancer multi-step process. This is referred to as field cancerization, a field of mutated epithelium containing various degrees of PMDs [14,15].

Frequently, field cancerization manifests in macroscopically invisible lesions and therefore represents an extremely challenging obstacle for predicting which patient will undergo the precancer-to-cancer transition. The serious limitations in dealing with these PMDs and highlighting the struggles with their nomenclature is our cluelessness about the risk and rate of their malignant transformation: the risk for malignization varies between 1–40% (!) depending on the study, with an average of about 9% [16]. The desperate state has been summarized by Aguirre-Urizar et al. [16]: “to date there are no pathognomonic factors or specific data that enable us to accurately predict which OLs [OPMDs] from a cohort may suffer malignant transformation.” This dismal state is reflected by the fact that, to this date, there is not a single FDA-approved chemoprevention treatment for HNSCC [17] despite a long tradition of attempts to develop such a treatment [18,19].

In this review, we summarize the current knowledge of the PMD stroma and its similarities and differences to the tumor microenvironment (TME), especially regarding how the PMD stroma has been neglected when it comes to grading PMDs for diagnostic purposes. Therefore, we will explore whether we know enough about these changes from PMD stroma to TME to be able to better understand the critical steps from precancer to cancer. We will further explore whether there are sufficient data on the PMD stroma so that it could serve as a putative target to prevent the precancer-to-cancer transition. As we will see, current knowledge on the PMD stroma is incomplete and requires substantial work before the enigma of the potential for malignant transformation of PMDs can be explained.

## 2. Materials and Methods

### 2.1. Search Strategy to Review the Topic of Stromal Changes in HNSCCs

We searched Pubmed and Google Scholar for literature on the topic of stromal changes in human HNSCC precancerous lesions using variations of “(stromal OR fibroblast OR immune) (premalignant OR precancer OR precancerous OR dysplasia OR leukoplakia OR erythroplakia OR OPMD) (HNSCC OR OSCC OR ‘oral cancer’ OR tonsil OR tongue OR larynx).” The variations occurred mainly in the first search feature, e.g., by replacing “(stromal OR fibroblast OR immune)” with the cell type or feature of premalignant lesions we were reviewing. We focused almost exclusively on human lesions, protein expression data, and in situ expression analyses.

### 2.2. Ultraplex Immunofluorescence Stainings and Analysis

**Figure 1:**
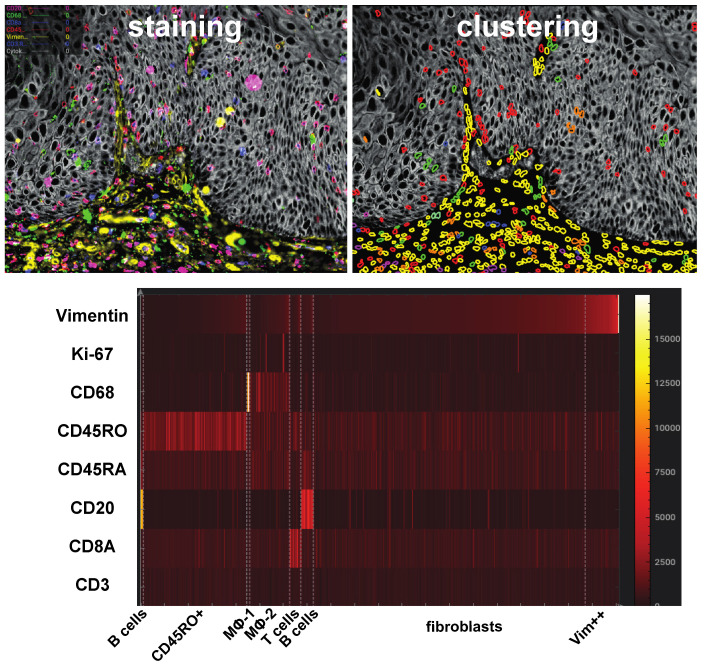
Example of a “simple” multiplex immunofluorescence staining using 18 antibodies on a Miltenyi MACSima instrument. For segmentation and clustering, the MACS iQ View software version 1.1 was used. In this example of an analysis of an HNSCC dysplastic lesion, stromal cells were clustered using Kmeans and the stromal markers VIM, CD8A, CD3, CD68, CD204 (MSR1), CD20, CD271 (NGFR), CD147 (BSG), CD45RA, and CD45RO, plus markers such as histone H1, TOM22, Ki-67, BCL2, JNK2, and ANXA1. On the **top left**, the actual image is shown with pan-cytokeratin in gray, CD3 and CD8a in shades of blue, vimentin in yellow, CD68 in green, and CD45RO in red. Using only non-epithelial cells for Kmeans, several clusters were defined (**upper right**) and characterized in a heat map (**lower** image). One CD8a+, two CD20+, two CD68+, a CD45RO+, and a VIM++ cluster were defined. Similar clusters were obtained using UMAP.

The tissue sample shown as an example in Figure 1 was obtained from a commercial source (TissueArray.Com LLC, Derwood, MD, USA). Our research on this human sample is regarded as non-human research because we obtained de-identified tissue slides that have already been in existence and were not collected specifically for our study. The tissue was provided as a formalin-fixed, paraffin-embedded (FFPE) tissue section that was processed by routine xylenes-based deparaffination, alcohol, and water washes. For tissue unmasking, we used a 98 °C water bath for 20 min in TEC buffer (20 mM Tris, 20 mM EDTA, 10 mM Sodium citrate, pH 9) as recommended by Miltenyi Biotec. The slide was then processed in Miltenyi’s MACSima automated staining and imaging platform according to the manufacturer’s protocols (Miltenyi Biotec, Bergisch Gladbach, Germany) using Miltenyi antibodies suitable for human FFPE tissue. Image analysis was performed with the Miltenyi MACS iQ View software version 1.1 (Miltenyi Biotec).

## 3. The Stroma

Some of the first quantifications of immune infiltrates using antibodies in HNSCC and its precursor lesions have established an association between mononuclear cell infiltrates with the transition from normal to cancer [20]. Earlier studies have found similar results [21].

Since then, a clearer picture has emerged from using single cell RNA sequencing (scRNAseq) (reviewed by Stampe et al. [22]). The drawback of these scRNAseq studies has been the lack of precursor lesions in them and the loss of spatial information. A key finding of scRNAseq is that although tumor cells vary from patient to patient and even show intratumoral heterogeneity, the stromal cell components are similar in all sequenced tumors. This suggests that there are no “patient-specific subpopulations” of stromal cells [23]. In the Puram et al. dataset, more than half of the cells extracted from HNSCCs were non-tumor cells (2215 malignant and 3363 non-malignant). Similar results were obtained by Peng et al. [24], though in this publication, the percentage of tumor cells per sample varied between 0.16% and 94%.

According to Puram et al. and Peng et al., the two main classes of stromal cells in the tumors were T cells and fibroblasts. In Puram et al., the circa 1000 T cells represented about 4 clusters (Tregs, T-helper, cytotoxic T cells, and exhausted T cells). The number of exhausted T cells varied considerably between patients and may serve as an indicator for immunotherapy success. The circa 1500 fibroblasts clustered in three groups (ACTA2+ myofibroblast-like cancer-associated fibroblasts [CAFs], ECM-producing CAFs, and “resting” CAFs). The remaining circa 800 cells represented endothelial cells, macrophages, mast cells, B cells, dendritic cells, and myocytes.

In Peng et al., the main stromal cell type was T cells (ca. 41% of all stromal cells), followed by macrophages (18%), fibroblasts (17%), and endothelial cells (8%). The rest of the stromal cells consisted of B cells, monocytes, and mast cells. T cells were represented by the following subtypes in order of their numbers: T-helper cells, cytotoxic T cells, exhausted T cells, Tregs, and NKT cells.

The question is, how do these cell populations contribute to precancer and the transition from precancer to cancer? Is there a PMD stroma that indicates the risk for malignant transformation? In the following paragraphs, we will summarize our (limited) knowledge of individual cell types in PMDs to shed light on how a better understanding of precancerous stromal changes may inform novel interventions to stop the progress towards cancer.

## 4. Activated Fibroblasts and Myofibroblasts

Normal head and neck tissues contain virtually no myofibroblasts. In general, these cells are associated with tissue damage [25]. Accordingly, myofibroblasts were first described as cells in wound granulation tissue [26]. In tumors, myofibroblasts are considered to be the major driver of the so-called “stroma reaction” [27].

A frequently used myofibroblast marker is ACTA2 (alpha smooth muscle actin) [28]. However, other cells express ACTA2, especially in blood vessels (mural cells, i.e., vascular smooth muscle cells and some pericytes). This can complicate the analysis of myofibroblasts and has been pointed out in a meta-analysis of ACTA2+ myofibroblasts in oral precancer [29]. These issues are not restricted to ACTA2 but are true for several “fibroblast” markers, of which most lack specificity.

The ACTA2+ myofibroblast population has been intensively investigated in HNSCC and PMDs (reviewed by Custódio [30]). The first study to specifically address ACTA2-positive myofibroblasts in HNSCC was by Zidar et al. in 2002 [31]. The core finding of the publication, that the emergence of myofibroblasts is tightly linked to invasive cancer, has been replicated multiple times. The absence of myofibroblasts in PMDs suggest that they are “generated” specifically in response to cancer cell invasion. It also suggests that PMDs cannot be divided into precancer and non-precancer based on ACTA2-positive myofibroblasts.

However, a notable exception can be found in an OPMD related to areca nut consumption. Areca nuts contain alkaloids that are believed to be potent stimulators of fibroblasts [32]. Although still poorly understood, chewing areca nuts can produce oral submucous fibrosis (OSMF), which leads to difficulties opening the mouth and can eventually result in oral cancer [33]. Since the disease is characterized by scarring and tissue fibrosis, myofibroblasts can be associated with its presentation, especially in the advanced stages of OSMF [34].

Cancer-associated fibroblast (CAF) is often used as a synonym for ACTA2+ fibroblasts in the tumor stroma. However, ACTA2+ fibroblasts only represent one group of CAFs. As we have seen in the Puram et al. analysis of the tumor stroma, at least three groups of fibroblasts can be detected in HNSCCs that fit the description of CAFs, one being ACTA2+ CAFs. The nature of the other CAFs is not as well-defined.

Although ACTA2+ myofibroblasts are more or less absent from PMDs (with the exception of OSMF), non-ACTA2 fibroblasts, as well as any other fibroblast cell types that may exist in cancer, are rarely investigated. For example, in a lung cancer study, Lambrecht et al. [35] defined 52 stromal cell types, of which there were seven fibroblast clusters. Six of the fibroblast clusters were from cancer tissue, and one was associated with normal tissue. This raises the question if any of these ACTA2-low or ACTA2-negative fibroblasts may already be present in HNSCC precancerous lesions. Currently, we have no answer to this question.

One of the first studies to characterize fibroblasts derived from human HNSCC samples *and* dysplastic lesions, by Costea et al., has shed light on the diversity of CAFs that may go beyond ACTA2-positive and -negative populations, actually ignoring ACAT2 entirely [36]. Their findings indicate that fibroblasts from dysplastic lesions are different from CAFs, but they also can be different from normal fibroblasts. However, the differences between normal and dysplastic fibroblasts seem small and hard to pinpoint. Costea et al.’s CAF analysis provided a clearer view on the stromal fibroblast in cancer for the first time.

They identified two principal CAF populations, CAF-N and CAF-D, which both promote tumor growth in various assays, with CAF-N being more potent.

A minor drawback of the Costea et al. in vitro characterization study is the lack of “finding” the different fibroblast populations in human tissue samples. How much do these subtypes of fibroblasts that they have defined after in vitro cultivation resemble in vivo fibroblasts? What is their spatial distribution in tumors and dysplastic lesions? How do the subtypes reflect the local differences in epithelial cell biology? What are hardcore markers of those fibroblasts that can be used to detect them in tissue sections for diagnostic or prognostic purposes? Finally, why are there only two main CAF populations in HNSCC, while in other carcinomas, they were able to “mathematically” distinguish six CAF populations [35]? This may suggest that in some scRNAseq analyses, virtual cell types may emerge that do not truly exist as definable subtypes in vivo, or that in vitro, most of these CAF populations cannot be maintained. The verdict on this is still out.

However, the two subtypes of CAFs identified by Costea et al. from HNSCCs overlap very well with the main CAF populations identified by Puram et al. [23]. Their scRNAseq dataset supports the observation of two major CAF populations, CAF1/myCAF (markers: PDPN, FN1) and CAF2/iCAF (CXCL12, GSN), that are highly similar to Costea et al.’s CAF-D and CAF-N, respectively. The third population of fibroblasts is defined by high ACAT2 and TAGLN. Luo et al. referred to them as “myofibroblasts.”

In the same vein, Liu et al. used seven existing RNA expression datasets from a broad spectrum of cancers to define CAF populations. From their analysis, three main fibroblast populations crystalized, i.e., CAF1/myCAF (markers PDPN, FN1), CAF2/iCAF, and myofibroblasts [37]. Taken together, these data suggest that there could be an overemphasis on ACTA2-positive “CAFs,” and one can boil down CAFs to two main subtypes which do not exist in PMDs. This new view of CAFs has not manifested itself in PMD and HNSCC stromal analysis by in situ methods such as immunostainings.

Consequently, the only other fibroblast marker that has been studied in dysplastic lesions is CD34 [38]. CD34 stromal cells may represent resident stromal fibroblasts. During wounding or invasion of cancer cells, such CD34+ stromal cells may transdifferentiate into ACTA2+ myofibroblasts. Consequently, the loss of CD34+ fibroblasts accompanies the precancer-to-cancer transition [38]. Similar to ACTA2, CD34 is not a perfect fibroblast marker and requires diligence when performing the analysis.

Similar results have been obtained using RNAseq data. The dysplasia–cancer in situ (CIS)–HNSCC transition is tightly associated with the emergence of CAFs, based on a study on sinonasal SCC progression from inverted papilloma to CIS to SCC. The transition is defined by genes associated with the tumor micro-environment and ACTA2 as a key indicator for the involvement of CAFs [39].

However, beyond ACTA2 and CD34, fibroblast populations have basically been ignored in PMDs.

## 5. T Cells

The role of T cells in the transition from dysplasia to cancer has not been reviewed in a while [40], despite the fact that T cells are most likely the dominant lymphoid cell infiltrate in PMDs and cancer. Since then, several studies have deepened our insights into T cell infiltration into PMDs. The main tools have been antibodies against CD4 (T-helper), CD8 (cytotoxic T cells), FOXP3 (Treg), CD43 (also known as SPN, memory T cell), CD45RO (memory T cell), CD25 (Treg), and PD1 (also known as PDCD1, exhausted T cells). Furthermore, studies using mRNA gene expression analysis [41] allowed a subclassification of OPMDs into two main classes. One class is characterized by “immunological” pathway activation, while the second class is characterized by high levels of EGFR expression. In a similar study by Llorens et al. [42], a major differentially expressed gene set associated with OPMD, summarized with the phrase “activation and regulation of the immune response,” seems to represent mainly T cells. Therefore, these mRNA-based analyses suggest that the main drivers of the immunological class of OPMD were T cells and, to some extent, monocytic and myeloid cell types. No differences were determined in neutrophil, endothelial cell, or fibroblast populations.

The main finding of studies using immunostainings of T cells can be summarized by a dramatic increase in T cells upon the acquisition of invasive behavior, reminiscent of the findings of ACTA2+ myofibroblasts [43]. However, in contrast to myofibroblasts, T cells are already abundant in PMDs when compared to normal or hyperplastic mucosa [44,45,46]. Similarly, Öhman et al. found an increase in CD3+, CD4+, and CD8+ T cells in leukoplakias with dysplasia compared to leukoplakias without dysplasia, and a further increase in SCCs [47]. Gannot et al. described an increase in CD4+ and CD8+ T cells from hyperkeratosis to dysplasia and cancer [48]. In general, the number of CD8+, CD4+, and FOXP3+ T cells increases with the grade of the dysplastic lesion and is highest in SCCs [44,45,49,50,51]. A similar result was obtained by Kouketsu et al. [52], associating progression from precancer to cancer with the infiltration of CD25+ and FOXP3+ Tregs.

An earlier study by Kambič et al. used SPN and CD45RO to detect putative effector memory T cells which behaved similarly to CD4 and CD8 T cells: their number and infiltration increased from normal to dysplasia and cancer [53]. However, Stasikowska-Kanicka et al. [54] and Piva et al. [55] have reported a decrease in CD8+ T cells from leukoplakia with dysplasia to oral cancer.

Despite some inconsistencies, the overall trend for T cell involvement is an increased presence from normal to dysplasia to cancer. This seems to be true for CD8-, CD4-, and FOXP3-positive populations.

The status of γδ T cells has been completely ignored. These T cells express a different T cell receptor than CD8+ and CD4+ T cells and can perform important functions in anti-tumor immunity [56]. Although γδ T cells comprise only a minority of T cells, the lack of knowledge about changes in their distribution in PMDs highlights the incomplete record we have on the PMD stroma.

Another T cell related protein, PD1 (also known as PDCD1), has been studied in the context of HNSCC precancer; however, its specific expression on T cells has not yet been determined in this cell population.

## 6. NK Cells

One definition of NK cells in flow cytometry is using CD3^neg.^/NCAM1(CD56)^+^/CD16^+^, upon activation of CD69^+^ and/or CD25^+^ or CD107A^+^ [57]. Such a detailed strategy to clearly define NK cells has never been used in HNSCC or HNSCC precursor tissue sections and therefore it is often unclear how accurate the definition of NK cells has been in the studies we discuss here. Additional single markers have been used, such as CD57 (also known as B3GAT1), to “define” NK cells [53,58], but a publication spending significant efforts to clearly identify NK cells in human PMDs is still pending.

Based on CD57, Kambič et al. [53] found little evidence of a major contribution of NK cells to the progression from normal to cancer. However, the number of CD57+ cells increased from normal to dysplasia and cancer. The overall finding on laryngeal lesions, their immune infiltrate, and the effects on the epithelium prompted Kambič et al. to state that the immune infiltrate was “most likely not an effective defense in preventing hyperplastic lesions from becoming malignant” [53]. This interpretation suggests that at the stage of precancer, the immune system does not appear to fight off aberrant cells.

NCAM1/CD56 has previously been used by Bondad-Palmario [46] with similar results in oral leukoplakia. A mild but statistically significant increase in CD56+ cells was observed from oral hyperplasia to severe dysplasia. No epithelial infiltration was observed. The same trend has been described for CD56+ in leukoplakias, with mild or absent dysplasias to leukoplakias with severe dysplasia [58].

In conclusion, a limited number of studies have explored changes in NK cell numbers in the transition from normal to dysplasia and cancer using mainly NCAM1/CD57 as the marker of choice. It seems a more detailed study is warranted to better define NK cells and their contribution to oral carcinogenesis.

## 7. Macrophages

Another abundant cell population in HNSCCs is macrophages. Although not as prominent as T cells [23,43], they are nevertheless believed to play crucial roles in tumorigenesis [59].

Macrophages (MΦ) are often divided into two phenotypic or functional subclasses called M1 and M2 [60,61]. Tumor-associated macrophages (TAMs) are generally associated with an M2-like subtype with immune-suppressive and pro-angiogenic properties. Consequently, an increased involvement of such M2-like TAMs is believed to be associated with poor prognosis [62]. However, most studies on macrophages in PMDs have not subcategorized macrophages based on this M1 and M2 polarization, but instead use a pan-macrophage marker such as CD68.

We have evaluated 16 studies that used macrophage/monocyte markers in PMDs and cancer [43,44,46,48,49,51,52,53,54,63,64,65,66,67,68,69] using antibodies against CD68 (pan-MΦ), CD86, CD80 (both M1-MΦ), CD163 and CD204 (both M2-MΦ), and CD14 (monocyte/macrophage marker).

The main theme for macrophages is their increase in abundance in tumors. Less clear is their role in PMDs. Lahav et al. [43] (CD68) and Stasikowska-Kanicka et al. [54] (CD163) reported no increase from normal and low-grade dysplasia to high-grade dysplasia. However, more detailed studies such as those by Dong et al. [44] and by Bondad-Palmario [46] show a significant increase from normal to dysplasia and with the grade of dysplasia using CD86 as a marker. Similar results were obtained by Lu et al. [67] and Sun et al. [51] with CD68 as a marker. Their findings have been confirmed using CD163 as a marker by Wang et al. [68]. Ye et al. [65] used both CD68 and CD163 and observed a significant gradual increase in macrophages from normal to cancer. A similar approach was taken by Kouketsu [52] using two M2-associated markers CD163 and CD204. Both of these markers showed a gradual increase from normal to low-grade and high-grade dysplasia. However, neither CD163 nor CD204 further increased significantly in the investigated cancer samples when compared to high-grade dysplasia. Seminerio et al. [64] observed the same trend, but using CD68, they only observed an increase in macrophage epithelial infiltrate, with progression from normal to low-grade dysplasia, high-grade dysplasia, and cancer. No differences were observed in the stromal compartment of PMDs. However, stromal increases in CD163+ macrophages were seen by Yagyuu et al. [49].

Using a less specific marker, i.e., CD14 (monocyte/macrophage), Gannot et al. [48] could observe a trend of an increased number of CD14+ cells from normal to PMDs, but the findings were not statistically significant and the CD14+ cells were actually reduced in tumor samples.

Few studies have explored the subtype of macrophages in PMDs. The most detailed attempt has been by Mori et al. [69]. Using CD68 (pan-MΦ), CD80 (M1 MΦ), CD163 (M2 MΦ), and a simple binary model of macrophage phenotype (M1-CD80 versus M2-CD163), they could only observe a significant increase in CD163+ cells in dysplastic leukoplakias, while CD68 and CD80 remained the same. However, the authors claim that CD163+ macrophages are actually more like M1 macrophages in the context of the progression of leukoplakias towards cancer. Emerging evidence suggests that tumor-associated macrophages (TAMs) and most likely some PMD-associated macrophages may not fit into the static model of M1 and M2 phenotypes, and that TAMs may represent unique populations of macrophages [70]. This also suggests that CD163+ macrophages may compromise the bulk of precancer and cancer-associated macrophages. For example, CD80+ macrophages are rare in OSCC [69].

In summary, although macrophages have been studied intensively, what is missing is a detailed study applying a comprehensive set of markers, e.g., CD68 and CSF1R as pan or M0 macrophage markers, CD86, CD80 and CD64 as M1 markers, and CD206, CD163 and CD204 as M2 markers. The complexities and phenotypic flexibility of macrophages may require such a comprehensive approach to better understand macrophage biology in the transition from precancer to cancer.

## 8. B Cells

Although changes in B cell infiltration are on par with changes in myofibroblasts and represent some of the most striking changes in the stroma of HNSCCs, only a few studies have explored their role [20,43,46,48,53,71].

One of the first studies on B cells was by Migliorati et al. in 1986 [20]. They used an anti-CD20 antibody and determined that the CD20+ cell number was increased with the severity of leukoplakia and in OSCC. Kambič et al. also rarely detected CD20+ B cells in PMDs [53]. This finding still holds true over 30 years later and has been recently confirmed by Lahav et al. [43] and Lao et al. [71]. Lahav et al. also used an anti-CD20 antibody and found a massive increase of B cells in tumor samples. However, the authors concluded that B cells may remain uninvolved in the tumorigenic process and that most of them remain in the periphery of the tumor. Lao et al. used an antibody against CD19 and could not detect B cells in PMDs, and in OSCCs, they only found sporadic infiltration into the tumor. Most B cells aggregated in the form of a “Crohn’s-like reaction” [72]. Such “germinal center-like structures” or tertiary lymphoid structures have been reported in oral leukoplakias with severe dysplasia and some with moderate dysplasia [46]. When taking into account these “Crohn’s-like reactions,” Bondad-Palmario detected an increase in B cells from hyperplasia to dysplasia [46].

On the other hand, Gannot et al. described a considerable increase in B cells from mild to severe dysplasia using a CD19/20 Pan-B cell cocktail [48].

In summary, B cells have been understudied when it comes to the transition from precancer to cancer and basically only two markers have been applied to detect them, i.e., CD19 and CD20.

## 9. Langerhans Cells and Dendritic Cells

Langerhans cells (LCs) are normal intra-epithelial residents of squamous epithelia and form a cordon of surveillance to detect foreign epitopes within the suprabasal cell layers. In contrast to most other cell types we have discussed so far, LCs may not increase steadily from normal to dysplasia and cancer. Since LCs are already relatively abundant in the normal epithelium, only one study observed an increase. Based on CD1A positive cells, Kindt et al. determined that the number of LCs increased in the intra-epithelial and stromal compartments of tumors compared to dysplastic lesions [73]. Intra-epithelial LC numbers increased from normal to dysplasia, although the increase was modest. Two other studies reported an increase of CD1a+ LCs from hyperplasia to dysplasia in both the epithelium and the stroma, and from normal to dysplasia [46,74], respectively. However, Upadhyay et al. did document a drop of intra-epithelial LC numbers from mild/moderate to severe dysplasia and to HNSCC [74].

These complex and often small differences in LC numbers and location may account for the fact that most other studies do not report an increase, but a decrease of LCs in HNSCC and its PMDs compared to normal epithelium [75,76]. Low numbers of LCs may be associated with poor prognosis [77,78]. However, although Costa et al. reported a decrease in CD1A+ LCs from normal to cancer, they observed a significant increase in CD83+ cells from normal to leukoplakia and cancer [76]. In contrast to CD1A, CD83 is associated with mature LCs but can also be expressed by other cell types (macrophages, B cells, monocytes) which generally increase with the progression towards HNSCC.

Only one study explored dendritic cells beyond LCs, i.e., plasmacytoid dendritic cells using CD303 (CLEC4C) as a marker. This study by Pellicioli et al. [79] confirmed a reduction of CD1a and CD83 LC population in cancer compared to precancer, but an increase of CD303 positive cells in cancer. As with many of the results described here, it is unclear whether CD303+ cells promote or inhibit tumor growth. Their number and increase are modest, suggesting that CD303+ cells may play a minor role in HNSCC.

In conclusion, changes in LC and dendritic cell numbers and location are complex during the progression from normal to HNSCC. The meta-analysis suggests a trend towards reduced presence of intra-epithelial LCs in tumors compared to dysplasia, and particularly compared to normal.

## 10. Granulocytes: Neutrophils, Eosinophils

### 10.1. Neutrophils

The observation that tumors may represent never-healing wounds should bring attention to neutrophils. Neutrophils are the most abundant cells in the bloodstream and represent some of the earliest responders to wounding. In line with other inflammatory cells, there is an increase in granulocytes (CEACAM8+) and neutrophils (myeloperoxidase, MPO+) in HNSCC tissues. This increase is associated with a poor prognosis [80]. Despite this correlation with advanced tumor stage and prognosis, the fact that tumor-associated neutrophils appear to come in two flavors, N1 and N2, which are either anti-tumorigenic (N1) or pro-tumorigenic (N2) [81], is rarely discussed. However, it is unclear whether N1 and N2 truly represent neutrophil phenotypes or, rather, describe different neutrophil maturation states in mice. Furthermore, it is unclear whether these findings in mice have relevance to the human condition [82]. Staggering differences in mouse and human neutrophil biology may make it more difficult to truly compare neutrophil function in tumorigenesis [82].

Another important factor to consider is the choice of neutrophil markers used in studies and their specificity for neutrophils. Concerns have been voiced about the nature of N2 neutrophils and whether they may represent myeloid-derived suppressor cells [82,83,84].

Furthermore, an important product of neutrophils, neutrophil extracellular traps (NETs), have not been explored in PMDs. NETs can be produced either from live neutrophils or represent the end stage of NETosis that results in the death of the neutrophil [85]. Cell-free DNA (cfDNA), chromatin, and proteases are the main components of NETs that help to form extensive networks to “trap” microorganisms. Beyond their existence, production, and components, the contribution of NETs, and, by extension, neutrophils, to HNSCC formation and progression is unclear [86].

### 10.2. Eosinophils

The infiltration of eosinophils into tumor tissue has been recognized at least since 1896 [87], and this observation eventually resulted in the term tumor-associated tissue eosinophilia (TATE). Despite this longstanding recognition, that eosinophil infiltration occurs in some tumors, the role of eosinophils in the tumorigenic process is poorly understood (reviewed adequately by Grisaru-Tal et al. [88]). However, there is a clear trend that TATE is associated with a favorable outcome in many cancers, including HNSCCs [88,89,90].

Although there is evidence that TATE is a favorable prognostic indicator, for PMDs, the verdict is less clear. Deepthi et al. [91] provide evidence that eosinophil infiltration based on Congo Red stain increases from dysplasia to OSCC. Madhura et al. [92] confirmed these findings and show a steady increase of eosinophils from normal to severe dysplasia. However, this finding could not be reproduced by Jain et al. [93].

The limitation of all these studies is the use of eosinophilic dyes rather than specific molecular markers for eosinophils, such as eosinophil peroxidase (EPX) or PRG2 (also known as major basic protein, MBP). The reliance on histological stains rather than molecular markers when it comes to eosinophils may relate to their high RNAse content. As discussed by Grisaru-Tal et al. [88], this may have resulted in a serious underrepresentation of eosinophils in various RNA-based datasets, and especially in scRNAseq data.

In summary, the contributions of eosinophils to the inflammatory processes during tumorigenesis are still unclear. TATE appears to be rare in HNSCC, but eosinophils may still perform important anti-tumor functions that have not been explored enough. Use of protein markers such as EPX and PRG2 may help overcome some hurdles.

## 11. Mast Cells

The sole intention of studying mast cells in HNSCC and its PMDs has been to better understand neoangiogenesis during invasion. Indeed, mast cells tend to be associated with blood vessels in cancer. From this point of view, mast cells are just packets of pro-angiogenic factors.

Mast cells are granulated cells which can release, e.g., histamines and heparin from their granules. A main tool to stain mast cells is toluidine blue, which mainly binds to glycoaminoglycans such as heparin.

Upon release, these granular components can mediate increased blood vessel permeability. However, mast cells are not a monolithic cell type and come in at least two subtypes, M(T) and M(TC) cells, which are rich in tryptase or tryptase and chymase, respectively [94]. However, none of these subtypes have been studied in the context of HNSCC, and the toluidine blue has been the main “marker” to detect mast cells. Overall, mast cells have been relatively poorly characterized in any human tumor and the contradicting reports on their association with patient prognosis suggest that they may be a much more complex cell population than expected (reviewed by Majorini et al. [95]).

In HNSCC and PMDs, mast cells follow a general trend similar to other immune cells in the majority of studies—a gradual increase from normal to dysplasia to cancer—and are generally associated with microvessels [96,97,98,99,100,101,102]. However, even this basic analysis of mast cells produced different results in other groups [103,104,105,106]. The reason for the differences in these studies is unclear. Few studies used anti-tryptase antibodies to determine mast cell identity and these studies could not detect an increase in mast cells in cancer tissues (e.g., Teófilo [106]).

In conclusion, the changes to mast cells during the progression from normal to tumor and their role during this progression are still unclear despite a plethora of studies on the topic.

## 12. The Endothelium

As we have seen in the investigations on mast cells and their association with neovascularization, microvessel density (MVD) appears to increase from normal to cancer. Microvessels mediate the blood–tissue exchange to supply oxygen and nutrients and remove CO_2_ and waste. Microvessels consist of capillaries and postcapillary venules and are composed of endothelial cells and pericytes. In tumors, microvessels can also be associated with smooth muscle cells.

Generally, MVD is highest in cancer samples (e.g., in one of the earliest studies on this issue using antibodies [anti-PECAM1] [107]). Using VWF, López de Cicco et al. detected a mild increase in MVD from normal to dysplasia, while in tumors, MVD, on average, almost doubled [108].

Of course, not all studies agree on this trend [109]. Also using PECAM1, Arora et al. could not detect an increase in MVD from dysplasia to SCC [110]. A review on the topic by Forster et al. from 2017 [111] also showed various outcomes when mainly focusing on data on hotspot measurements of MVDs in normal, dysplastic and tumor tissues. The trend is clear with generally a strong increase from normal to dysplasia and another increase to cancer, but even for normal samples, the MVD counts varied from an average of 19 to 88 MV/mm^2^. These differences highlight the difficulties in analyzing and quantifying microvessels.

Since this review by Forster et al. [111], the increase in MVD has been documented by an astonishing plethora of studies [99,100,102,106,112,113,114,115,116,117].

### 12.1. The Conundrum of Neovascularization in Precancer

What is striking from all of these studies that show a significant increase in vascularization already in dysplasia is the fact that dysplastic lesions rarely appear to outgrow the “magic” 2 mm^3^ that then require new blood vessels to form in order to avoid necrosis. (Folkman himself uses the number of 0.03 mm^3^ [118], which is more in line with in vitro findings on avascular spheroids and the limit of oxygen diffusion: non-necrotic spheroids grow up to 400 µm in diameter.) Therefore, the increase in MVD in dysplastic lesions appears unwarranted when using the 2 mm^3^ number due to a lack of size and invasiveness. However, any increase in size and depth of a lesion compared to normal may already pose a supply problem for the tissue and require neovascularization. Perhaps an increase in MVD merely describes the formation of extra microvessels with poor functionality. Tumor vasculature is generally characterized by its poor ability to transport oxygen and nutrients and therefore may lead to the formation of even more microvessels. Therefore, the question is whether MVD, by itself, is sufficient to characterize lesion vascularization.

However, the simplest explanation for these increased numbers of microvessels seems to be an already elevated expression of angiogenic factors such as VEGF in PMDs.

### 12.2. VEGF and Hypoxia

The first published report of increased precancer VEGF expression stems from 1997. Denhart et al. [119] documented elevated VEGF mRNA expression, but only in high-grade dysplasia and OSCC. Similar results were obtained by Sauter et al. using an anti-VEGF antibody. However, they did not observe an abrupt increase in high-grade lesions [120]. This more gradual increase in VEGF expression has been confirmed by another immunohistochemical analysis of VEGF expression from normal to HNSCC [121].

However, in regard to VEGF, the same question arises as for MVD: why is VEGF elevated if we assume that its expression is triggered by hypoxia and/or the “angiogenic switch” [122]?

Surprisingly few studies have addressed hypoxia in PMDs. This may be due to the lack of absolutely trustworthy markers and measurements. For example, expression of carbonic anhydrase 9 (CA9) is regarded as an immunohistochemically useful marker to evaluate hypoxia. CA9 has emerged as a prognostic biomarker for HNSCCs [123] and has also been evaluated in PMDs. Dysplasia-free leukoplakias did not express CA9, but a subset of dysplasias did [124]. In general, CA9 expression increases with severity of the dysplasia and is highest in carcinoma in situs [125,126]. CA9 expression in dysplasia is moderately associated with the risk for malignization. The combination of CA9 with p53 overexpression and age reached excellent predicative values for the progression of a dysplastic lesion to cancer [127]. An independent study by Zhang et al. [128] reached a similar conclusion, suggesting that CA9 expression in dysplastic lesions is a strong predictor of a patient’s likelihood of developing a malignant tumor.

### 12.3. Hypoxia and Metabolic Reprogramming

Another indicator of early changes in hypoxia-related gene expression comes from studies on metabolic reprogramming in PMD lesions of squamous epithelia. Cervical PMDs already exhibit metabolic changes that are reminiscent of the changes seen in cancer [129], i.e., indications of the Warburg effect. The authors could detect increased glucose consumption and lactate production in PMDs compared to normal mucosa. Accordingly, the expression of the glucose transporter GLUT1 (SLC2A1) is steadily increased from normal to HNSCC [130,131].

A major transcriptional regulator of many of the above-mentioned genes (CA9, SLC2A1) is HIF1A. Activation of HIF1A is mediated by hypoxia. If there is indeed hypoxia in PMDs, as indicated by the so-called hypoxia marker CA9, then one should expect that HIF1A is coordinating this upregulation. Several studies have suggested such an upregulation of HIF1A in PMDs [132].

Interestingly, there is evidence for “hypoxia-associated oxidative stress” occurring already in PMDs [133] as well.

### 12.4. The Angiogenic Switch as an Opportunity for Diagnosis

In summary, the “angiogenic switch” appears to be an early event in HNSCC formation. Folkman already recognized and summarized this paradoxical finding in 1996: the “angiogenic switch … becomes activated during the early stages of tumor development” [118]. In HNSCC, this is not only supported by the observed increase in MVD in PMDs in most studies, but also by the increase of angiogenic factors, mast cells, and signs of hypoxia and hypoxia-related gene expression.

Based on the above-described findings, a non-invasive application to improve identification of patients at high risk for malignant conversion of dysplasia to cancer could be Narrow Band Imaging (NBI).

The changes in the vascularization that accompany progression of lesions towards cancer may be pinpointed by NBI endoscopy. NBI endoscopy basically visualizes blood vessels in superficial lesions [134] and may identify lesions of interest in the cancerization field [135]. Consequently, NBI endoscopy results match MVD data well [116].

As a precautious note here, efforts to correlate hypoxia and hypoxia-related gene expression in HNSCC have failed miserably [136,137,138], suggesting that the same may be true for PMDs. Determination of hypoxia-related gene expression is performed on FFPE tissue samples that may have undergone changes before fixation due to tissue handling and time between resection and fixation. Such artefacts are rarely discussed. However, it seems well established that hypoxia, even in PMDs, exists and determines treatment outcome (e.g., [139]). The hypothesis is that increased size of PMDs drives hypoxia that, in turn, induces an HIF1A response with increased but poor-quality vascularization, and CA9, VEGF, GLUT1 expression. This may change the PMD microenvironment (e.g., acidification through lactate), driven by a switch from oxidative phosphorylation to glycolysis, with the associated advantages of providing fast access to ATP and key metabolic building blocks.

The microenvironmental acidification may lead to an immune suppressive environment, which we will explore next.

## 13. Immune Checkpoint Markers

As we have seen so far, an overabundance of stromal changes is already present in precancerous lesions. However, it is unclear whether many of the inflammatory changes are associated with anti-cancer or pro-cancer functions. A hallmark of cancer is to avoid elimination by the immune system, i.e., using immune suppression. Is this also a faculty that PMDs have mastered? A recent review [140] has addressed many aspects of the topic and we want to highlight the immune checkpoint system that is at the heart of most clinical efforts.

One of the main immune regulatory systems is based on PDCD1 (PD1) and its ligand CD274 (PD-L1). The first study to explore this system in the progression from precancer to HNSCC, by Malaspina et al. [141], observed a striking increase in PDCD1-positve CD4+ and CD8+ T cells in cancer tissues compared to leukoplakias. Assuming the central role of the PDCD1-CD274 axis in immunosuppression and T cell exhaustion, it is surprising that a meta-analysis of studies on CD274 expression in HNSCCs could not detect a correlation with prognosis [142]. However, the authors observed a high variability between the studies they analyzed.

On the other hand, a meta-analysis of CD274 expression in PMDs [143] confirmed an increase in CD274 in the transition from normal to PMD and cancer. Again, “high heterogeneity and moderate quality suggest that further studies with larger sample size” will be necessary to clarify the status of immunosuppression in PMDs.

Since then, several studies have confirmed this overall trend of induction of epithelial CD274 expression in PMDs, especially in lesions at high risk for malignant conversion [144]. A recurring theme can be summarized by a “significant heterogeneity of PD-1 and PD-L1 staining between fields within the samples” [144]. This may be a major reason for the inconsistencies between studies. A more elaborate study on several immune markers also detected an increase of CD274 in PMDs [145], which confirms the overall trend [146].

The question is, how is CD274 upregulation, especially in cancer cells, achieved? And could knowledge about the mechanisms of its upregulation help define the transition from precancer to cancer? Of all the suspected transcriptional regulators of CD274 [147], a main regulator appears to be IRF1 [148]. Indeed, loss of IRF1 expression in vitro in cancer cell lines results in a significant reduction of CD274 expression [149]. In HNSCC tissue samples based on TCGA data, CD274 shows a significant co-expression with IRF1, interferon gamma, and STAT1 [150]. Unfortunately, no publication has explored the regulation of IRF1 in human PMDs of the head and neck region.

On the other hand, PDCD1 (PD1), the receptor for CD274, appears to be mainly expressed in lymphocytes in PMDs and SCC lesions [151]. As with many other features we have explored in this review, immunohistological levels of PD-L1 and PD-1 already show a clear indication of immunosuppression, even in PMDs. Consequently, a key conclusion the authors drew from their studies was that “an abnormality of the PD-L1/PD-1 pathway might already be present at a certain point before premalignant lesions become invasive or malignant” [151].

## 14. Crossing the Border: The Critical Step of Invasion into the Stroma and the Emergence of the “True” Tumor Stroma

Based on the stromal changes we have discussed so far, few changes appear to be cancer specific, and many of the hallmark cancer-associated stromal changes occur already in PMDs.

The boundary between the epithelium and the stroma is defined by the basal lamina. The basal lamina and its extracellular matrix represent a highly specialized aspect of the stroma and are the first barrier for tumor cells to freely migrate beyond their normal location into non-cancerous tissue. Crossing this boundary is the defining moment of invasion and malignancy.

The main components of the basal lamina underlying the epithelia of the head and neck region are collagen IV, laminins, perlecan (HSPG2), and nidogen. Interruption in the continuous staining pattern of all these markers has been documented in PMDs and HNSCCs. However, it is unclear whether the disturbance of the basal lamina is initiated in the epithelium or if it is due to the presence of strong inflammation which may lead to local degradation of the basal lamina (e.g., [152]).

Ultrastructural analyses have found that the basal lamina is already disturbed in PMDs and hardly exists anymore in OSCC [153,154]. Detailed IHC studies of the basal lamina also indicate that its disruption occurs already in PMDs, especially in severe dysplasia and carcinoma in situ CIS [155,156]. However, these precancerous disruptions also appeared to correlate with inflammation and may represent secondary events potentially caused by inflammatory cells [157]. In line with this is the finding that the major component of the basal lamina, collagen IV, is relatively normal in most dysplastic lesions but can be disrupted in CIS. Collagen IV levels overall were reduced in cancer samples [158]. However, the expression of perlecan (HSPG2) was already lost from the basal lamina starting in dysplastic lesions [159] and is strongly reduced in cancer [160].

### The Paradox of Hemidesmosomal Protein Upregulation in Precancer and Cancer

What is astonishing is that although the basal lamina tends to become interrupted in cancer, proteins associated with a main structure that adheres cells to the basal lamina, the hemidesmosomes, are actually overexpressed in cancer and some PMDs. COL17A1, ITGA6, and ITGB4 are epithelial basal cell markers that directly contact the basal lamina components (mainly laminins) and they all show increased expression in HNSCCs [161,162]. The same is true for laminins. For example, laminin LAMC2 is, like hemidesmosomal markers, overexpressed in HNSCC/OSCC and may be a marker for high-risk PMDs advancing towards cancer [163,164].

Interestingly, increased keratinocyte expression of certain laminins is observed during wound healing and may promote keratinocyte migration. Again, the concept that cancer is a poorly healing wound seems to come to fruition. An almost general observation in cancer, including HNSCC, is that some laminins are overexpressed, and this overexpression is associated with a more aggressive phenotype. Often, these laminin overexpressing cancer cells appear at the tumor front and indicate invasive behavior. A comprehensive review on the topic by Rousselle and Scoazec highlights this peculiar finding of increased laminin expression in a broad spectrum of cancers [165].

In summary, disruption of the basal lamina can be observed in a subset of PMDs and crossing the basal lamina appears to be the hallmark event in truly remaking the stroma to the advantage of the tumor cells. In contrast to several immune cells that can cross the basal lamina with ease, e.g., through small pores within the basal lamina [166,167,168,169], these pores appear far too small for keratinocytes. The acquisition of a tool set to remake the basal lamina may be an obligate requirement for invasion. If this tool set is key to understanding the invasion and the remaking of the stroma, the question is: what are the genetic drivers that bestow cancer cells with this tool set to manipulate the basal lamina?

## 15. Epithelial Changes That May Drive Cancerous Stromal Remodeling: Which Genetic Drivers May Run the Show of Stromal Invasion and Remodeling?

Although many mutations and expression changes have been associated with HNSCC compared to the normal squamous epithelium, the changes within the epithelial cells that induce stromal remodeling and, eventually, invasion are surprisingly poorly defined. Several review articles have tried to summarize the overall sequence of events that lead up to invasive cancer, but no “invasion and stromal remodeling master gene” has been identified [170,171,172,173].

An obvious theme is that genetic changes, especially copy number aberrations, increase from normal to dysplasia and to cancer [174]. However, which changes drive invasive behavior and stromal remodeling is unclear. Some rough clues come from studies “narrowing” down genomic areas of interest that may be involved in the dysplasia-to-cancer transition. For example, Noutoumi et al. [174] have suggested that losses on chromosomes 3p14-21 and 5q12-22 are associated with the acquisition of invasive capabilities. However, these regions contain dozens of genes and likely several regions that are involved in tumorigenesis. A narrower definition by Yoo et al. [175] mentions two loci on chromosome 3p, i.e., 3p14.2 and 3p25, that may represent the transition from dysplasia to cancer. A similar region had been implicated by Bhosale et al., suggesting that, “3p26.3, 3p21.1 … were identified as regions of copy number-dependent gene down-regulation.” It is clear that even for chromosome 3p, there is little consensus on which gene(s) may drive the formation of invasive cancer and whether changes in these genomic regions are relevant for invasiveness [176].

Another approach to define the genetic basis for invasion through the basal lamina into the stroma is to look at the key “ingredients” that are associated with invasion and what master regulators may regulate these “ingredients.” Although these ingredients should be easy to define, it is neither clear if the cancer cells themselves initiate the breakdown of the basal lamina (BL) nor whether they recruit cancer-associated fibroblasts (CAFs) or inflammatory cells to do the job. This chicken-and-egg question may also differ from epithelium to epithelium. In the breast, it is believed that the transition from ductal carcinoma in situ (DISC) to carcinoma requires CAFs.

However, to this day, it is debatable whether the sequence is DISC-CAFs-BL breakdown-cancer or DICS-BL breakdown-cancer-CAFs. The term “occult” seems adequate to describe the molecular biology behind the acquisition of invasive behavior and the sequence of events that lead to invasion [177].

### Invasive Behavior as a Key Cancer Therapy Target

Targeting this critical step has been a promising target in cancer therapy, but unsurprisingly, due to a lack of its molecular underpinnings, it has been completely unsuccessful (e.g., MMPs, [178]). Furthermore, MMP-independent invasion may occur either with some help from fibroblasts [179] or by plain force [180]. A forceful entry into the stroma may require huge quantities of ATP and glucose [180]. Therefore, markers that indicate increased metabolic activity may be useful to identify areas of cell invasion. However, there seem to be no data available linking ATP production and consumption with invasion or the invasive front.

In summary, the molecular definition of the critical step to invasive cancer is still abysmal. Beyond the association of a “damaged” basal lamina and altered expression of hemidesmosomal and matrix metalloproteinase markers, little is known about the drivers of the defining moment in making cancer.

In the end, it appears to be a chicken-and-egg situation: do cells first acquire the capability to push through the basal lamina and then remodel the stroma, e.g., by recruiting CAFs, or do the epithelial cells first acquire the ability to recruit CAFs and other cells, which then open up the basal lamina and allow cancer cells to invade [179]? There is some evidence for both scenarios and the expression data suggest that severe manipulation of the basal lamina and the appearance of CAFs seem to be the only markers absent in PMDs and present in cancer. Moreover, once CAFs emerge, the TME experiences a whole series of fundamental changes, including profound changes in stiffness.

## 16. Conclusions

A wealth of genetic, expression, and biochemical data have been accumulated on HNSCC and PMDs. However, no useful picture has emerged on how and which precursor lesions transition into the malignant state. Therefore, no therapy has been successfully implemented to suppress this transition.

We have sieved through the publication record on the state of the tumor stroma and the stroma of PMDs in hopes of generating a synthesis from the wealth of often conflicting data. Unfortunately, we have been able to extract only a few kernels of wisdom from all the data; i.e., that the vast majority of stromal changes associated with cancer can already be detected in subsets of PMDs, with the notable exception of CAF induction, and mastering the breakthrough of the BL. This means that only ACTA2 CAFs are a decent unique feature of HNSCC and that CAFs are basically absent from PMDs. Hypoxia and expression of CA9 and the emergence of immunosuppressive features (CD274 expression) appear to be meaningful markers associated with the risk for malignization (Table 1).

A limitation of our review of the literature is the general lack of a clinically relevant correlation between the analyzed PMDs and the formation of SCCs. As mentioned earlier, field cancerization may produce a plethora of lesions with a wide range or precancer potential. Therefore, most studies rely on a very rough approximation of malignization risk of PMDs. A solution to this problem might be the use of substantially larger cohorts of clinically and histologically better-defined lesions. Quantity and quality of the investigated material most likely must be improved.

To explain the inconsistencies within the published data on the PMD and cancer stroma, there are few answers. HNSCCs are complex tumors originating in an anatomically diverse area. Such complexity may produce local precancerous phenotypes. Furthermore, there are two major forms of oral cancer (HPV+ and HPV−) and not unmixing these two types may have skewed the results of many studies.

Another limitation of most studies included in this review is the reduction of the complexities of tumor–stroma interactions to one cell type. Ignoring these complexities may miss important features of PMDs that may make them look more like SCCs and may predict their malignant potential. In PMDs, the complexities of the epithelial–stroma interactions, and especially the interactions between different stromal cell populations, are poorly defined. Complex systems are more than the sum of their parts, and therefore, before being able to reduce this complexity, we should appreciate the diversity of the PMD stroma and disentangle it. Currently, most studies have only analyzed one or two cell populations, while only a minority has started to explore the idea of multiplexing their approach, i.e., studying multiple cell populations with multiple markers simultaneously. The technologies to perform such highly multiplexed analyses while maintaining spatial information at the single cell level are by now well established, commercialized, and refined constantly [181,182,183,184,185,186,187] (Figure 1).

A highly multiplexed approach may also solve some of the issues regarding the specificity of many markers and subtyping of certain cell types. Several of the markers discussed here are most likely mis-identifying cell populations due to their limited specificity. In this case, an increase in the number of the investigated markers, their quality, and specificity may further improve the correct identification of cell populations within PMDs and HNSCCs.

Because PMDs in the head and neck area are complex and small, currently the only reasonable approach to dive into their complexities while preserving spatial information and the ability to perform histopathological analysis is a multiplex immunofluorescence approach. Once data are available that use the full power of these highly multiplexed immunofluorescence imaging methods to deep-profile the transition from precancer to cancer, we can start to untangle the complexities of the stroma in relation to changes in the epithelium.

As an example, all the markers that have been used in the vast majority of studies on PMDs of the head and neck region, summarized in Table 1, can be run in a medium complex multiplex immunofluorescence stain in an automated instrument within 24 h on multiple tissue samples and analyzed quasi simultaneously. Figure 1 offers a small glimpse of how the future of stromal analysis could look. Figure 1 shows a relatively low-plex immunofluorescence staining, the detection of epithelial and stromal cell population, and the detangling of stromal population using clustering tools, such as Kmeans, or nonlinear dimensionality reduction tools, such as UMAP or t-SNE. However, even with a more detailed study of each individual sample, the obstacle of sample quality remains. How well established is the relationship between the investigated sample and cancer formation in the patient? The hope is that a deeper characterization of each PMD will produce a defined subpopulation of PMDs that are tightly associated with malignization.

## Figures and Tables

**Table 1:** Summary of the findings and relevance of the different stromal components in the progression from normal to HNSCC.

Cell Type	Markers	Trend	Conclusion
Fibroblasts/CAFs	ACTA2, CD34	Abrupt appearance in SCC	Marker for SCC
T Cells	CD4, CD8, FOXP3, CD43, CD45RO, CD25, PDCD1	Increase from normal to dysplasia to cancer	Already abundant in precancerous lesions
NK Cells	CD57, NCAM1	Little evidence for association with progression from normal to cancer	More detailed study needed to better define NK cells
Macrophages	CD68, CD86, CD80, CD163, CD204, CD14	CD68, CD86, CD163, CD204: Significant increase from normal to dysplasia CD14: Findings not statistically significant	Missing detailed study applying comprehensive set of markers
B Cells	CD19, CD20	Rarely detected in PMD lesions	Understudied. May remain uninvolved in tumorigenic process
Langerhans Cells	CD1A, CD83	Reduced presence of intra-epithelial LCs in tumors compared to dysplasia/normal	Changes in cell numbers/location complex during progression from normal to HNSCC
Neutrophils	CEACAM8, MPO	Increase in HNSCC tissues	Contribution to HNSCC formation/progression unclear
Eosinophils	Congo Red and histological stains	Steady increase from normal to severe dysplasia	Contribution during tumorigenesis unclear. Specific molecular markers (EPX or PRG2) should be studied
Mast Cells	Tryptase, histological stains	Gradual increase from normal to dysplasia to cancer	Role during progression from normal to tumor unclear
Endothelium and Hypoxia	PECAM1, VWF, VEGF, CA9, HIF1A, NBI endoscopy	Significant increase in vascularization already in dysplasia; elevated expression of angiogenic factors in PMD lesions	Hypoxia present in precancerous lesions
Immune Checkpoint	CD274, PDCD1	Induction of epithelial CD274 expression and PD1 expressed in lymphocytes in PMD lesions	Immunohistological levels of PD-1 indication for immunosuppression in PMD lesions
Basal Lamina	Ultrastructure, COL4A1, COL17A1, HSPG2, laminins, ITGB4, ITGA6	Already disturbed in PMD lesions	Unclear if cancer cells, CAFs, or inflammatory cells initiate breakdown

## Data Availability

Data relating to Figure 1 are available on request. The data presented in Figure 1 are part of a larger cohort that will be published separately.
